# Motivation to test, treat, and report malaria cases: a quantitative assessment among private sector providers in the Greater Mekong Subregion

**DOI:** 10.1186/s12936-022-04108-7

**Published:** 2022-03-09

**Authors:** Morgan Brown, Paul Bouanchaud, Kemi Tesfazghi, Saysana Phanalasy, May Me Thet, Hoa Nguyen, Jennifer Wheeler

**Affiliations:** 1 Consultant, PO Box 99, Stratton, OH 43961 USA; 2 Population Services International, 1120 19th St NW, Suite 600, Washington, DC 20036 USA; 3 Population Services International Laos, T4 Road, Unit 16, Donkoi Village, Sisattanak District, Vientiane Capital, Lao People’s Democratic Republic; 4 Population Services International Myanmar, No.16 Shwe Gon Taing Street 4, Yangon, Myanmar; 5 Population Services International Vietnam, VinaFor Building, 127 Lò Đúc, Phạm Đình Hổ, Hai Bà Trưng, Hanoi, Vietnam

**Keywords:** Malaria, Malaria elimination, Private sector, Provider motivation, Confirmatory factor analysis

## Abstract

**Background:**

Accurately testing, treating, and tracking all malaria cases is critical to achieving elimination. Ensuring health providers are able and motivated to test, treat, and report cases is a necessary component of elimination programmes, and particularly challenging in low endemic settings where providers may not encounter a large volume of cases. This study aimed to understand provider motivations to test, treat, and report malaria cases to better optimize programme design, adjust incentive schemes, and ultimately improve reporting rates while growing the evidence base around private providers in the Greater Mekong Subregion (GMS).

**Methods:**

With funding from the Bill & Melinda Gates Foundation, this study aimed to identify and validate distinctive subtypes of motivation among private sector providers enrolled in the Greater Mekong Subregion Elimination of Malaria through Surveillance (GEMS) programme, implemented by Population Services International. Quantitative questionnaires were administered electronically in person by trained enumerators to various provider groups in Myanmar, Lao PDR, and Vietnam. A three-stage confirmatory factor analysis was then conducted in STATA.

**Results:**

Following this analysis, a two-factor solution that describes motivation in this population of providers was identified, and providers were scored on the two dimensions of motivation. The correlation between the two rotated factors was 0.3889, and the Kaiser–Meyer–Olkin (KMO) measure of sampling adequacy was 0.93, indicating an excellent level of suitability. These providers, who are often assumed to only be financially motivated, engaged in malaria elimination activities because of both internal and external motivational factors that are independent of remuneration or financial gain. For all three countries’ data, significant covariances between the two latent variables for internal and external motivation were found. The models were found to be of adequate to good fit for the data across all three countries. It was determined that private sector providers, who were previously believed to be primarily financially motivated, were also motivated by personal factors. Motivation was also associated with key outcomes of importance to malaria elimination, such as reporting and stocking of tests and treatments.

**Conclusion:**

Maintaining or increasing provider motivation to test and treat is essential in the fight to eliminate malaria from the GMS, as it helps to ensure that providers continue to pursue this goal, even in a low incidence environment where cases may be rare and in which providers face financial pressure to focus on areas of health service provision. Establishing mechanisms to better motivate providers through intrinsic factors is likely to have a substantive impact on the sustainability of malaria case management activities.

## Background

Important gains towards malaria elimination have been achieved in recent years in the Greater Mekong Subregion (GMS). Although malaria cases and deaths have declined dramatically, the expansion of artemisinin resistance in the region is a growing threat to malaria control efforts. Thorough surveillance is critical to curbing the epidemic, and private sector contributions, while important, often go unreported [1, 2]. Established in 2016 and funded by the Bill and Melinda Gates Foundation, the Population Services International (PSI) Greater Mekong Subregion Elimination of Malaria through Surveillance (GEMS) programme aims to increase private sector engagement to accelerate progress towards elimination.

Motivation has been defined as, “the level of effort and desire to perform well” and is an important determinant of quality of care [3]. Motivation in an employment setting is defined as *“… a set of energetic forces that originate both within as well as beyond an individual’s being, to initiate work related behavior, and to determine its form, direction, intensity, and duration*” [4]. Motivation has been associated with lower levels of staff turnover [5], higher retention, less job burnout, increased performance [6], and higher quality of care [7]. Further, motivated employees come to work more regularly, work more diligently, and are more flexible [8].

The evidence base on provider motivation in a malaria context is limited, particularly in terms of a robust, multi-country quantitative analysis. Existing studies tend to be qualitative in nature, conducted in the public, not private, sector, and largely focused on community health workers (CHWs) [9–11]. Within the malaria context, existing literature explores provider motivations for testing and treatment [12–14], but little is known regarding provider motivations for case reporting. There is also a dearth of literature for Plasmodium falciparum elimination contexts [15, 16].

Quantitative measurement of provider motivation involves defining motivation, a multidimensional construct. It must also consider the multiple components of motivation that influence behavior, and the context-specific language used to discuss motivation in different cultural settings. Furthermore, employment motivations can differ in both conceptualization and measurement between different provider subgroups. Direct measures are typically derived through measurement scales within a survey or through qualitative methods. Indirect measures of motivation can be derived through surveys or qualitative methods via experimental games or observations of behaviour [17]. Further, where measuring motivation has been attempted, it has been focused on the public and community sectors.

In 2000, Bennett, Franco, Kanfer and Stubblebine developed a specific tool to measure the determinants and consequences of public sector health worker motivation in developing countries, which was then used in a three-part study of health worker motivation in hospitals in Jordan and Georgia [18]. The tool encompasses a number of motivational and performance categories, theoretical constructs, and scales.

In 2017, Lohmann et al. [19] developed a psychometric scale to measure motivation composition. The scale was grounded in the self-determination theory (SDT), a theory introduced in the 1980’s as a general framework of human motivation. The SDT captures a generalized measure of motivation toward work and identifies five dimensions of motivation that can be placed along a continuum from extrinsic (motivation to attain or avoid a consequence that is maintained by rewards/punishment) to intrinsic (motivation stemming from the enjoyment of a task).

Much progress has been made in the GMS, particularly during the last five years, to reduce the malaria burden. In Lao PDR, the number of confirmed malaria cases decreased between 2012 and 2019 by 80% [20]. Vietnam has fewer than 5000 confirmed cases per year, mostly concentrated in three provinces: Binh Phuoc, Dak Lak, and Gia Lai [21]. In the past decade, the number of reported malaria deaths in Myanmar has dropped steadily year by year from 1707 in 2005 to just 19 in 2018 (a 99% reduction over 10 years). The incidence of reported malaria has fallen by 85% since 2012 (from 9.94 per 1000 population in 2012 to 1.46 per 1000 population in 2018) [20].

Engaging the private sector is necessary to achieve malaria elimination in the GMS, as a significant proportion of the population first seeks health care within that sector. The preference for private sector providers is likely related to accessibility and perceptions of quality and flexibility in prescribing medicines when compared to the public sector [22]. However, none of the six countries in the GMS collect complete case data from private sector points of care such as pharmacies, clinics, shops and private hospitals [23]. As a result, national policy makers lack access to a complete malaria case data set to inform programme strategies and interventions.

In 2015 and 2016, cross sectional outlet surveys identified low availability of malaria diagnostic testing (Cambodia, 75%; Lao PDR, 94%; Myanmar, 75%) and poor access to first-line treatment in the private sector (Cambodia, 70.9%; Lao PDR, 40.8%; Myanmar *P. falciparum* = 42.7%, *P. vivax* = 19.6%) across the GMS [24, 25]. Between 2015 and 2019, the GEMS programme received funding from the Bill & Melinda Gates Foundation to support national malaria control programmes (NMCPs) in Cambodia, Lao PDR, Myanmar and Vietnam to capture private sector data by engaging private providers in malaria case management, generating private sector malaria case data, and integrating these data into national surveillance systems. The GEMS established network has increased access to quality case management in Cambodia, Lao PDR, Vietnam and Myanmar, where use of the private sector remains high [22]. PSI-supported providers, funded from the GEMS project, detected between 1.8 and 18% of the national caseload in each of the four countries in 2019 [25].

Achieving malaria elimination will require a concerted effort from actors across the public and private sectors. Private sector involvement in malaria surveillance is a relatively recent development. This study sought to understand the motivations of private sector network providers to test, treat, and report malaria cases and determine barriers to quality performance. Quality performance within the GEMS programme defined as achieving 80% or more during quality assessment visits. The quality of care is monitored routinely using standardized checklists developed in collaboration with the national programme and according to international benchmarks for quality malaria service provision. The present study aims to identify and validate different underlying subtypes of motivation among private sector providers enrolled in the GEMS programme by using a three-stage confirmatory factor analysis followed by a two-factor solution. Similar modelling approaches have previously been applied to understand determinants and barriers to vaccine coverage [26] and malaria control measures [27]. How these subtypes of motivation differ by provider characteristics was then examined, in addition to whether they are associated with intentions and outcomes related to malaria service provision. These analyses will allow us to develop recommendations that influence policies on the role of the private sector in national malaria elimination strategies.

## Methods

### Study population

Formal healthcare providers (for the purposes of the study are providers supported by the GEMS project and permitted to operate in the national context) served as the sampling frame for this study and were eligible for inclusion if they were actively enrolled in the GEMS malaria programmes and consented to participate. GEMS works with different provider types in each country, thus specific provider types varied accordingly.

In Myanmar, three types of providers were sampled from the PSI supported networks: POs, ICMVs, and SUN Network providers. In Vietnam, three provider cadres were targeted for this study: clinics, pharmacies, and FMCGs (CMCs were excluded from this study). In Lao PDR, clinics and pharmacies were included in this study. In Lao PDR, these providers are largely identical and have similar educational and training requirements and responsibilities.

A random sample of providers, stratified by type of provider, was drawn from each country’s list of providers who met the study inclusion criteria (Table 1). A sample size calculation for the number of providers needed to estimate key measures was used with a precision of ± 7.5%. Providers within each stratified group were selected using a simple random sampling. In Myanmar, providers were selected using systematic sampling.

**Table 1:** Incentives by channel

Channel	Country						
	Lao PDR	Myanmar			Vietnam		
	PPM	PO (AMTR)	ICMV (CHSP)	Sun	Clinic	Pharmacy	FMCG
Channel Definition	Formal Providers (pharmacies and clinics)	Private outlet: comprised of Non-formal private providers (general retailers, sundry shops and itinerant drug vendors)	Community-based health service providers	Formal private providers (general practitioner clinics)	Formal private providers (general practitioner clinics)	Formal licensed pharmacists	Fast moving consumer goods outlets comprising of general retailers and selected sundry shops
Malaria services provided	Testing, treat and report	Testing, treat and report	Testing, treat and report	Testing, treat and report	Testing, treat and report	Test and refer	Test and refer
Incentives: USD/month per provider; or max. possible if performance based	$ 40	Maximum USD 5	Maximum $ 10	Maximum $ 16	No monetary incentive (Promo items only, ~ $20)	No monetary incentive (Promo items only, ~ $5)	No monetary incentive (Promo items only, ~ $5)

### Survey instruments

For this study, a quantitative survey instrument was developed, borrowing from previously tested measures of motivation [18, 19] and incorporating additional questions with input from in-country expert teams with the dual aim of ensuring programmatic relevance while generating robust measures. Respondents were asked a range of questions about their motivations for participating in PSI’s malaria programme, measured using five-point Likert-type scales. All motivation questions were identically written and administered across the three countries, while some programmatic questions varied.

Questionnaires were administered in person by trained enumerators and responses were captured electronically. This study was approved by the PSI Research and Ethics Board and local review boards in each of the three countries (Myanmar MM—PSI REB #26.2018; Lao PDR LA—Local IRB #2018.69.MP and Vietnam VN—Local IRB #462/2018/YTCC-HD3.) All participants provided informed consent and data were deidentified prior to analysis. The provider motivation module is shown in Annex 1.

### Analysis

Provider motivation was conceptualized to be a multidimensional construct and, given the dearth of previous research into provider motivation in the GMS, a large number of survey items hypothesized to relate to provider motivation were included in the data collection tool. To first establish that the items used in the questionnaire did in fact pertain to the different elements of motivation suggested in the literature, [17] an exploratory factor analysis (EFA) was conducted followed by a review of the substantive content of the questions to validate that the questionnaire items were measuring two constructs, intrinsic and extrinsic motivation, in the Myanmar data. Next, confirmatory factor analysis (CFA) was used to test construct validity in the Lao PDR and Vietnam data. Factor loadings were then used to calculate weighted scores, which were used in subsequent analyses. The analysis proceeded in the following stages:

Stage 1: The analysis was conducted initially on the Myanmar dataset. EFA was used to explore the underlying structure of the correlations between the survey items and to develop a parsimonious set of provider motivation questions relevant to this context. A two-factor model was fitted based on an examination of scree plots and factor eigenvalues. Of the original 32 items pertaining to motivation in the dataset, 16 items with communalities greater than 0.5 were retained. Using maximum likelihood estimation, and promax (oblique) rotation of the factors, a simple solution was achieved (i.e. each item loading onto only one factor). The use of oblique rotation reflects our expectation that the two motivation subtypes are correlated.

Stage 2: A CFA model was developed, using the structural equation modelling (SEM) command in Stata, with two latent variables and item loadings reflecting the simple solution found in the EFA model. This model was first fitted for the Myanmar dataset. The same model specification was then applied separately to the Lao PDR and Vietnam datasets to validate the proposed structure. The results and Satorra-Bentler (adjusted for non-normality) goodness of fit statistics are detailed in the results section below.

Stage 3: For each of the three CFA models (Myanmar, Lao PDR and Vietnam), predicted Bayes scores were calculated (with zero mean, and unit variance) for each provider on both latent factors using the SEM postestimation *predict, latent* command in STATA. Further analyses were conducted on these scores to examine how these two dimensions of provider motivation were associated with a series of background demographics and outcome variables of interest. Significance testing was performed on these analyses using oneway ANOVA, simple linear regression, or t-tests according to variable type.

## Results

Respondents were majority female (53% Myanmar, 77% Lao PDR, and 54% Vietnam), averaging 44, 48, and 43 years of age in Myanmar, Lao PDR, and Vietnam, respectively (Table 2). In Myanmar respondents had an average of 5 years of experience working as a provider, whereas in Vietnam providers had an average of 15 years of experience. In Lao PDR, this question was not asked. Instead, providers were asked how long they had worked in the PSI programme, with an average of 6 months reported. Levels of education varied widely across countries.

**Table 2:** Provider sample size

Country	Provider type	Sample size
Lao PDR	Formal Clinic/Pharmacy Providers	96
Myanmar	Sun Quality Health (SUN) Network Providers	132
	Integrated Community Malaria Volunteers	150
	Private outlets	134
Vietnam	Formal Clinic Providers	96
	Pharmacies	134
	FMCGs	13

### Factor analysis

Following the three-stage process described above using data from Myanmar, EFA identified a two-factor solution that describes motivation in this population of providers. Annex 3 shows the unrotated and rotated factor loadings for a bidimensional model for provider motivation with loadings greater than 0.4 indicated in bold. The correlation between the two rotated factors was 0.3889, and the Kaiser–Meyer–Olkin (KMO) measure of sampling adequacy was 0.93, indicating an excellent level of suitability.

The face validity of the two factor solution rests on the theoretical model for motivation that identifies internal and external motivation as key constructs. Examining the questionnaire items that are associated with each of the two factors in the EFA confirms that internal and external motivation are the two key dimensions of provider motivation in Myanmar. The positive correlation between internal and external motivation dimensions confirms our assumption that both represent different dimensions a larger concept of “provider motivation” in this context.

**Fig. 1:**
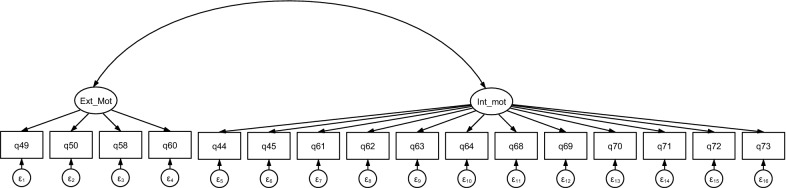
final confirmatory factor analysis path diagram

As the second stage in the analysis, a confirmatory model was estimated for the Myanmar data (Model 1, Table 3). Figure 1 below shows this model. The same bidimensional structure was then applied to the Lao PDR (model 2) and Vietnam (model 3) datasets. The model coefficients, *P*-values, covariance between latent variables, and goodness of fit statistics are shown in Table 3. Recognizing that financial motivations were predicted to be important in the literature, a further confirmatory factor analysis was conducted, adding a third latent construct for financial motivation, measured by three financial motivation-related items in the questionnaire. These items were only asked to two of the three provider types in Myanmar who receive financial compensation (model 4, Table 3).

**Table 3:** Sample characteristics for the three countries

		Myanmar	Lao PDR	Vietnam
	N	416	126	243
Gender	Female	53%	77%	54%
	Male	47%	23%	47%
Age	Mean (years)	44.5	48.5	43.2
	s.d	14.4	12.2	11.9
Time working as a provider*	Mean (years)	5.1	0.7	15.4
	s.d	4.8	0.5	11.3
Education	Monastery or Other	29%	3%	27%
	High School	25%	15%	7%
	Some college	3%	58%	30%
	Bachelor’s Degree	40%	20%	30%
	Masters or above	3%	4%	5%

^*^Time as provider was not collected in Lao PDR—this variable measures time in PSI program

### Loadings and country comparison

Four variables constitute the construct of external motivation in the original Myanmar analysis (model 1), and this was replicable for the Vietnam dataset (model 2), with all coefficients of a similar magnitude and statistical significance in both countries. In applying this model structure to the Lao PDR provider data (model 3), however, it was determined that one coefficient for the statement, “Because my reputation depends on it” was not significantly associated with the latent construct. Furthermore, a second coefficient for “It brings pride to my family to know that I’m contributing to malaria elimination” had a small loading. This suggests that the external motivation construct may look different or was not fully captured for providers in Lao PDR. The latent construct of internal motivation, modelled originally in the Myanmar dataset, was stable across both additional countries, with statistically significant coefficients of a comparable magnitude for Lao PDR and Vietnam. For all three countries’ data, positive, significant covariances between the two latent variables for internal and external motivation were found. The covariance was higher in Lao PDR and Vietnam than in Myanmar.

### Goodness of fit

Various measures of goodness of fit for structural equation and CFA models are proposed in the literature. Several are reported here in line with recommended best practices in SEM modelling. The models were found to be of adequate to good fit for the data across all three countries. The chi-squared test statistic, while indicative of good fit, is also sensitive to violations of its assumptions and may not be a very good indicator of model adequacy, particularly with smaller sample sizes [28]. To correct for sample size sensitivity, the chi-squared to degrees of freedom ration may additionally be considered. Values below 3 indicate model adequacy, with lower values indicating better fit. All three models presented here have adequate fits according to this measure. RMSEA is less sensitive to sample size than chi-squared. Values below 0.05 indicate close fit, between 0.05 and 0.08 fair fit, and between 0.08 and 0.1 mediocre fit [29]. Comparative fit index (CFI) values greater than 0.9 are considered to indicate good fit. In this analysis, both Lao PDR and Myanmar attain this criterion. Finally, the standardized root mean squared residual (SRMR) is a standardized measure of the difference between observed and predicted correlations, with a value of less than 0.08 indicating good fit. Models for both Lao PDR and Myanmar achieved this level, with Vietnam exceeding the cutoff only slightly.

To understand whether provider characteristics are associated with different levels of internal and external motivation, scores were predicted on the two dimensions of motivation for all providers in the three country’s datasets (Tables 4, 5, 6). These predicted scores were then used in subsequent analyses to understand how the two motivation subtypes vary according to characteristics of providers.

**Table 4:** CFA models for Myanmar, Lao PDR and Vietnam, showing standardized coefficients and significance levels

Motivation type	Item	Model 1		Model 2		Model 3		Model 4	
		Myanmar		Lao PDR		Vietnam		Myanmar AMTR and CHSP only	
		Coef	Sig	Coef	Sig	Coef	Sig	Coef	Sig
Measurement model									
External	Because my reputation depends on it	0.500	***	0.022	ns	0.773	***	0.523	***
	Because I receive appreciation for doing it	0.652	***	0.402	***	0.745	***	0.581	***
	It brings pride to my family to know that I am contributing to malaria elimination	0.787	***	0.161	*	0.490	***	0.778	***
	It is a source of pride to participate in the franchise malaria programme	0.593	***	0.638	***	0.469	***	0.559	***
Internal	Because the program is interesting	0.557	***	0.495	***	0.549	***	0.538	***
	Because it is extremely important for my patients	0.524	***	0.418	***	0.544	***	0.476	***
	I value the feedback about the effectiveness (e.g., quality and quantity)	0.619	***	0.563	***	0.492	***	0.536	***
	My franchise malaria programme-related job duties, requirements, and goal	0.665	***	0.417	***	0.588	***	0.591	***
	Participating in the SQH Franchise malaria program gives me a feeling of accomplishment	0.722	***	0.603	***	0.674	***	0.672	***
	I feel I am contributing to malaria elimination in my community and country	0.750	***	0.377	***	0.686	***	0.756	***
	Participating in the programme makes me feel like I’m doing something worthwhile	0.765	***	0.659	***	0.635	***	0.735	***
	There is satisfaction in a job well done	0.824	***	0.535	***	0.687	***	0.829	***
	I am satisfied with the chances I have to learn new things	0.870	***	0.741	***	0.711	***	0.891	***
	I am satisfied with the chances I have to accomplish something worthwhile	0.859	***	0.640	***	0.696	***	0.830	***
	I am satisfied with the chances I have to do something that makes me feel good a	0.717	***	0.573	***	0.642	***	0.752	***
	I am satisfied with the educational/training opportunities I get	0.751	***	0.447	***	0.695	***	0.737	***
Financial	Because of the financial benefits associated with it							0.921	***
	In order to be able to provide for my family financially							0.848	***
	In order to earn money/make a profit							0.563	***
Structural model									
External <—> Internal covariance		0.324	***	1.021	***	0.698	***	0.205	***
External <—> Financial covariance								0.234	***
Internal <—> Financial covariance								− 0.113	ns
Goodness of Fit									
Satora-Bentler adjusted GoF results (for non-normality)									
Model Chi2		246.945		127.883		307.334		267.679	
		< 0.001		0.049		< 0.001		< 0.001	
		103		103		103		149	
Chi2/df ratio		2.397524		1.241583		2.983825		1.796503	
RMSEA		0.058		0.044		0.091		0.053	
CFI		0.936		0.921		0.852		0.914	
SRMR		0.06		0.074		0.083		0.065	

^*^P < 0.05, ***P < 0.001

**Table 5:** Myanmar predicted external and internal motivation scores

Myanmar		Standardized mean scores	
		External motivation	Internal motivation
Provider characteristics			
Overall	Overall mean score	− 0.001	− 0.002
	SD	0.655	0.313
	Min	− 1.832	− 1.223
	Max	0.812	0.256
Gender	Female	0.050	0.029
	Male	− 0.058	− 0.037
Provider type	AMTR	0.210	0.030
	CHSP	− 0.017	0.085
	SQH	− 0.197	− 0.133
Region	Yangon	− 0.111	0.096
	Mandalay	− 0.029	− 0.114
	Sagaing	− 0.480	0.103
	Taninthar	0.378	0.084
	Shan	0.274	0.126
	Kachin	0.144	− 0.048
	Chin	0.059	− 0.017
Education level	Monastery	0.264	0.098
	High school	− 0.035	0.064
	Higher education	− 0.157	− 0.111
Provider age (years)	Regression coefficient:	0.003**	− 0.002*
Outcomes			
Attended PSI training	Yes	− 0.009	0.004
	Not yet	0.200	− 0.152
How willing would you be to continue to keeping records and sharing them with the government?	Extremely unwilling	− 0.118	− 0.133
	Not willing	− 0.128	− 0.019
	Neutral	− 0.292	− 0.278
	Willing	− 0.024	− 0.047
	Extremely willing	0.149	0.138
Imagine that all of PSI’s support for your practice ended tomorrow. How would you restock on commodities	I wouldn't restock	− 0.054	− 0.101
	Receive from the government	− 0.078	0.130
	Buy from another source	0.038	− 0.005
	Other	0.038	− 0.029
Time in programme (yrs)	Regression coefficient:	− 0.008	− 0.012***

Bold scores indicate significant difference within group at the 5% level (ANOVA); significance of regression coefficients = * = 5%; ** = 1%; *** < 0.1%

**Table 6:** Lao PDR predicted external and internal motivation scores

Lao PDR		Mean scores	
		External motivation	Internal motivation
*Provider characteristics*			
Overall	Overall mean score	0.000	0.000
	SD	0.017	0.225
	Min	− 0.059	0.798
	Max	0.014	0.185
Gender	Female	− 0.001	− 0.009
	Male	0.002	0.029
Region	Savannakhet	**0.006**	**0.081**
	Saravan	**0.006**	**0.802**
	Champasac	− **0.001**	− **0.005**
	Sekong	− **0.012**	− **0.169**
	Attapeu	− **0.008**	− **0.102**
Education level	High school	0.005	0.064
	Some college	− 0.001	− 0.011
	Higher	− 0.001	− 0.012
		Regression coefficient	
Provider age (years)	Regression coefficient:	0.000	− 0.001
Outcomes			
Willing to continue records/reporting if had to take reports to local health centre?	Yes	0.001	0.008
	No	− 0.002	− 0.025
Willing to continue records/sharing if could submit my phone or SMS	Yes	**0.002**	**0.027**
	No	− **0.005**	− **0.064**
Imagine that all of PSI’s support for your practice ended tomorrow. How would you …	I wouldn't restock	0.001	0.013
	Receive from the government	− 0.005	− 0.064
	Buy from another source	0.003	0.042
	Other	− 0.007	− 0.091
Time in programme (yrs)	Regression coefficient:	− 0.005	− 0.071

Bold scores indicate significant difference within group at the 5% level (ANOVA); significance of regression coefficients = * = 5%; ** = 1%; *** < 0.1%

Predicted scores for both latent constructs of motivation were standardized with zero mean and unit variance. Female providers in Myanmar had significantly higher average scores on the internal motivation scale than their male peers, while the opposite was found in Vietnam. No difference was found between female and male providers in Lao PDR.

Some geographic variation in motivation across all three countries was seen. External motivation in Myanmar was highest among providers in Tanintharyi Region and lowest in Sagaing Region, while internal motivation was highest in Shan State and lowest in Mandalay Region. In Lao PDR, providers in Saravan and Savannakhet scored highest on both internal and external motivation scales, while those in Attapeu and Sekong scored lowest. In Vietnam, significant differences by region were only seen for the internal motivation scale, where providers in Binh Phuoc scored highest, and those in Gia Lai lowest.

The education level of providers was significantly related to both dimensions of motivation in Myanmar and Vietnam, but the direction of effect differed between the two countries. In Myanmar, higher levels of education were associated with lower levels of both subscales of motivation. In Vietnam, providers reporting more advanced levels of education scored higher on both subscales of motivation.

The age of providers was significantly related to external and internal motivation scores in both Myanmar and Vietnam, with increased provider age associated with a small but significant increase in the standardized external motivation score in both countries, while internal motivation decreased with age in Myanmar and increased with age in Vietnam.

The measure of time spent in the PSI malaria programme was significantly related to providers’ internal motivation scores in Myanmar and Vietnam. In Myanmar, each additional year in the programme was associated with a small but significant decrease in internal motivation, while in Vietnam the relationship was positive.

The study also sought to understand what role external and internal motivation might play in predicting some key outcomes. In Myanmar and Vietnam, higher internal and external motivation scores were significantly associated with greater willingness to continue to maintain and share records with the government after the end of programme implementation. This implies that providers with higher motivation would continue to test, treat, and report malaria cases once financial incentives provided by the programme are withdrawn. This question was phrased differently in Lao PDR, where providers were asked if they would be willing to continue to share malaria case records by phone or SMS after the end of the programme. Here also for both internal and external motivation, significantly higher scores were associated with affirmative responses.

Finally, providers were asked what they would do to maintain malaria test and treatment stocks if the programme ended. In Myanmar those reporting that they would not restock had significantly lower levels of internal motivation than those with any other response. In Vietnam those reporting that they would not restock were found to have significantly lower scores on both internal and external motivation.

**Fig. 2:**
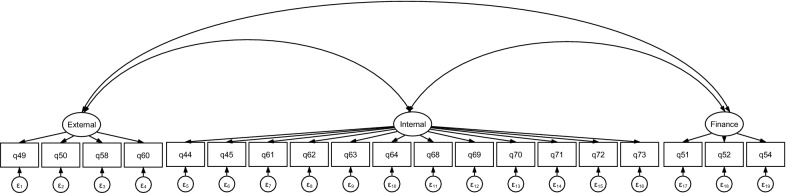
CFA path diagram for model including financial motivation

The inclusion of a third type of motivation related to financial benefits of being involved in the malaria programme resulted in a CFA model with adequate fit to the data (model 4 in Table 3, and Fig. 2). The coefficients and *P*-values for items loading on to internal and external motivation remained similar to the two-factor model (Models 4 and 1 in Table 3, respectively). The three finance-related items loaded positively on to the latent variable for financial motivation and all loadings were statistically significant at the 1% level. The structural part of the model suggests that when financial motivation is included, the positive and significant relationship between internal and external motivation persists. A non-negligible positive, statistically significant covariance between external and financial motivation types was also found. This means that providers who reported being more strongly motivated by external factors were also likely to be more strongly motivated by financial factors. The relationship between internal motivation and financial motivation was not statistically significant.

The predicted factor scores for the three motivation constructs (including financial motivation) are shown in Table 7. There was little statistically significant variation in financial motivation by background demographics. This suggests that the degree to which providers are motivated by money in their malaria testing and treatment activities is less related to age, education or gender than is the case for internal and external motivation. Likewise, financial motivation scores were not significantly related to the outcome of intention to continue to maintain records after the end of the PSI programme—one measure of programme sustainability. However, for the other outcome of source of supplies after the end of the programme, there was a significant relationship with financial motivation: providers who reported that they would not restock testing and treatments scored significantly higher on the financial motivation scale than their peers.

**Table 7:** Vietnam predicted external and internal motivation scores

Vietnam		Mean scores	
		External motivation	Internal motivation
*Provider characteristics*			
Overall	Overall mean score	− 0.008	− 0.004
	SD	0.636	0.427
	Min	− 1.276	− 0.804
	Max	1.505	1.133
Gender	Female	− **0.108**	− **0.079**
	Male	**0.108**	**0.082**
Provider type	Pharmacies	− **0.141**	− **0.111**
	Clinics	**0.214**	**0.161**
	FMCGs	− **0.273**	− **0.126**
Region	Binh Phuoc	0.109	**0.151**
	Dak Lak	− 0.031	− **0.003**
	Gia Lai	− 0.045	− **0.095**
Education level	High school	− **0.140**	**0.010**
	Some college	− **0.023**	− **0.072**
	Bachelors	**0.221**	**0.134**
	Masters	**0.379**	**0.355**
	Other	− **0.291**	− **0.162**
		Regression coefficient	
Provider age (years)	Regression coefficient:	0.008**	0.005**
Outcomes			
How willing would you be to continue to keeping records and sharing them with the government?	Extremely unwilling	n/a	n/a
	Not willing	− **0.246**	− **0.111**
	Neutral	− **0.295**	− **0.245**
	Willing	− **0.035**	**0.035**
	Extremely willing	**0.478**	**0.241**
Imagine that all of PSI’s support for your practice ended tomorrow. How would you restock …	I wouldn't restock	− **0.424**	− **0.309**
	Receive from the government	**0.171**	**0.145**
	Buy from another source	**0.035**	**0.021**
	Other	**0.613**	**0.307**
Time in programme (yrs)	Regression coefficient:	0.006	0.006**

Bold scores indicate significant difference within group at the 5% level (ANOVA); significance of regression coefficients = * = 5%; ** = 1%; *** < 0.1%

## Discussion

Ensuring that every suspected malaria case presenting to the public or private sector is tested and treated is critical to malaria elimination. To achieve this, motivating private providers to engage in national malaria response is key, particularly in areas where a significant proportion of the population seeks care in the private sector.

The measurement of healthcare provider motivation is difficult because it is a transitory construct that can be unidimensional or multidimensional and can be measured directly or indirectly [17]. Existing scales that measure health provider motivation use Likert-style psychometric measures consisting of multiple items to capture different dimensions of motivation [30]. These measures have mostly been developed for use in high-income countries and may not be appropriate for use context lower-and-middle income countries. This study applied previous measures of motivation to a diverse group of private providers in three countries in a malaria elimination context. The measures were developed from existing, validated tools developed to measure motivation of health care providers in low- and middle-income countries (LMICs). The scale developed by Lohman and colleagues in 2017 [19] is based on SDT and measures motivation composition (the relative contribution of different kinds of motivation to overall work motivation). SDT is recommended for supporting programmes to determine how motivation of different origins and characteristics contributes to overall motivation and to understand how differences in the dimensions of motivation are associated with outcomes of interest. Also included were measures from Bennet and colleagues [18], who developed scales drawing from published literature for constructs of motivational determinants, such as worker expectations, values/work ethic, work-related personality, and emotional personality in Jordan and Georgia. These measures were adapted for use within the GEMS malaria elimination programme in Vietnam, Myanmar, and Lao PDR. These adaptations included slight wording changes and removal of redundant items, which were later pretested prior to study implementation.

As found by other research in different contexts [31], the GEMS private sector providers, who were believed to be financially motivated, were also motivated by other personal factors. Motivations varied by key characteristics of providers and were predictive of outcomes of importance. Maintaining or increasing provider motivation to test and treat in malaria case management is essential in the fight to eliminate malaria from the GMS, as it helps to ensure that providers continue to pursue this goal, even in a low incidence environment where cases may be rare and in which providers face financial pressure to focus on areas of health service provision other than malaria case management.

These results demonstrate that in Myanmar and Vietnam (and to some extent in Lao PDR), provider motivation has two similar dimensions across countries. The relationship between internal and external motivation was also similar in all 3 countries, meaning that across these contexts, providers who have a higher level of external motivation are also likely to have a higher level of internal motivation. Examining how motivation varies by provider characteristics, results were highly country-specific. This is unsurprising—Vietnam, Lao PDR and Myanmar are very different contexts, with differently structured health systems and incentive systems for providers. While recent decades of underinvestment have weakened Myanmar’s public health system, significant improvements have been made during the last 5 years. Myanmar remains the most permissive environment for the private sector, and all PSI providers—including informal private outlets and volunteers—are able to test and treat *Plasmodium falciparum* and *Plasmodium vivax* malaria, including prescribing primaquine for radical cure of *P. vivax*.

Conversely, only private clinics are allowed to test and treat for malaria in Vietnam. In agreement with some provincial authorities, however, PSI has trained pharmacies, CMCs, and FMCG shops to test and refer malaria cases. These country-level differences may be reflective of the interplay between individual provider-level motivations and a unique relationship between organizational structure, culture, and societal culture [18].

When several important outcome variables in the analysis (willingness to continue to report cases after the end of the PSI malaria programme, and intention to acquire testing and treatment stocks post-PSI programme), were examined, it was found that in both Myanmar and Vietnam, higher provider internal and external motivation scores were associated with greater intent to continue these key aspects of malaria case management. This finding is timely as the programme phases out and the private sector provider networks transition to public sector oversight in each country. Finding ways to better motivate providers through intrinsic factors is, therefore likely to have a substantive impact on the sustainability of these activities during and after the implementation of this programme. It is thus possible to speculate that a fruitful approach to driving greater project sustainability may therefore lie in targeted recruitment or stratifying providers within each project country and developing interventions that will appeal to their core motivations. This challenges the conventional wisdom that providers only care about money and financial incentives are their primary source of motivation [32]. Indeed the analysis exploring financial motivation suggests it may only be quite weakly associated with external motivation in the model. Further research is needed to assess the degree to which these different dimensions of provider motivation may be change through time and, therefore, would have the potential to be increased through specific interventions. Similarly, it would be beneficial to know the degree to which this bidimensional structure of provider motivations might apply to other malaria service providers outside of the GMS. Further research should aim to identify interventions that increase internal and external motivations and to better understand the interplay between financial, internal, and external motivations and the value for money associated with interventions.

## Limitations

The findings may not be generalizable to the private sector as a whole, as the study sample consisted only of private providers that engaged in the PSI GEMS programme. Further, these results would need to be validated for use in other contexts and provider types. Participation in the PSI programme could have led to response bias among study participants. Self-report bias could also have influenced results. The analysis was based on Likert-scale responses, which are imperfect and treated as continuous variables, an approach commonly used in the literature [17], despite being discrete. Likewise, the use of Likert scale-type questions may violate the assumption of normality under a maximum likelihood estimation method for the CFA models. For the financial analysis, the model was only an adequate fit, perhaps because the sample size was smaller and the model was more complex. The findings are indicative but should be interpreted with caution. Finally, the providers’ reported outcomes were based on intentions, not observed actions, and further research might consider a longitudinal approach for examining associations between provider motivation and outcomes.

## Conclusions

Conventional wisdom has long held that private sector providers are primarily motivated by financial incentives. These results, however, show motivation to be multifaceted for this group. This study consistently identified two dimensions: internal and external motivation, across three contexts and different types of providers (ranging from informal to medical professionals). Providers chose to join PSI’s malaria programme for a variety of reasons, including commitment to serving their community, boosting their reputation, having access to professional development opportunities, and receiving commodities.

By understanding how motivation varies according to provider characteristics, malaria elimination programmes can better target continuing professional development, adapt incentive structures, and update training and routine communication with providers to build on factors that may improve internal and external motivation. These findings provide national programmes with the opportunity to better understand providers within their contexts which in turn can lead to better programme design leveraging appropriate incentive schemes and motivations in order to enhance provider performance and programme results in the local context.

Organizations need to look at providers not just as business owners and public servants, but also as nuanced actors with multiple sources of motivation. Individual characteristics are important to how private providers should be trained, recruited, and engaged to ensure long term success and sustainability. Future research should aim to better understand how motivation varies in different contexts and its effects on outcomes in the health system.

## Data Availability

The datasets used and analyzed during the current study are available from the corresponding author on reasonable request.

## Annex 1: Provider Motivation Questions

I participate in the PSI malaria program because:

**Table:**

Question	Motivational Domain
Because I enjoy the support and interaction with PSI	Intrinsic Motivation
Because the program is interesting	
Because it is extremely important for my patients	Integrated/identified regulation
Because I want to make a difference in people’s lives	
Because I want to serve my communities/relatives/neighbors	
Because it makes me feel good about myself	Introjected regulation
Because my reputation depends on it	
Because I receive appreciation for doing it	External regulation—social

**Table:**

Work is important because it enables one to be socially valuable	Social interaction
If I were known as an unreliable malaria provider, this would bring shame to my family	Shame
If I scored badly on a quality assessment conducted by PSI’s QI Officer, I would feel ashamed	
If everyone were to know that I did not report malaria cases, it would bring shame to my family	
It brings pride to my family to know that I’m contributing to malaria elimination	Pride
It is a source of pride to participate in the PSI Malaria program and receive PSI support	
I value the feedback about the effectiveness (e.g. quality and quantity) of my performance (i.e. when I’m doing well) I receive from PSI about my contributions to PSI’s malaria programme	Motivational properties
My job duties, requirements, and goals are clear and specific	
Participating in the programme gives me a feeling of achievement and accomplishment	
I feel I am contributing to malaria elimination in my community and country	
Participating in the programme provides adequate benefits to make it worthwhile	
My program responsibilities provide acknowledgement and recognition from clients	Job feedback
My program responsibilities provide acknowledgement and recognition from the community	
Participating in the programme makes me feel like I’m doing something worthwhile	Job preferences
There is satisfaction in a job well done	Desire for work achievement
I am satisfied with the chances I have to learn new things	Intrinsic job satisfaction
I am satisfied with the chances I have to accomplish something worthwhile	
I am satisfied with the chances I have to do something that makes me feel good about myself as a person	
I am satisfied with the educational/ training opportunities I get	Extrinsic job satisfaction
I am proud to tell others that I am part of this programme	Organizational commitment

## Annex 2: Univariate Means and Distributions

**Table:**

Variable	Obs	Mean	Std. Dev
*Myanmar (Full sample)*			
q49	416	3.204327	1.491851
q50	416	4.069712	1.250521
q58	416	3.754808	1.263949
q60	416	4.081731	1.085747
q44	416	4.682692	0.581057
q45	416	4.807692	0.462094
q61	416	4.533654	0.60426
q62	415	4.568675	0.589343
q63	415	4.590361	0.618478
q64	415	4.686747	0.527681
q68	415	4.653012	0.547356
q69	415	4.677108	0.512483
q70	415	4.684337	0.495507
q71	415	4.679518	0.487466
q72	415	4.575904	0.631995
q73	415	4.66747	0.560605
*Myanmar Financial Vars*			
q51	284	1.542254	0.914147
q52	284	1.552817	0.991497
q54	283	1.858657	1.29147

**Table:**

Variable	Obs	Mean	Std. Dev
*Lao PDR*			
q048	126	4.357143	0.80463
q049	126	4.595238	0.68327
q056	126	4.81746	0.598676
q059	126	4.84127	0.366883
q044	126	4.714286	0.48756
q045	126	4.785714	0.430946
q060	126	4.634921	0.545574
q061	126	4.777778	0.454117
q062	126	4.571429	0.720317
q063	126	4.896825	0.305401
q067	126	4.714286	0.51934
q068	126	4.722222	0.500222
q069	126	4.81746	0.463047
q070	126	4.738095	0.476295
q071	126	4.746032	0.55044
q072	126	4.825397	0.421034

**Table:**

Variable	Obs	Mean	Std. Dev
*Vietnam*			
q061	243	3.670782	0.921871
q062	243	3.600823	0.803607
q073	243	3.864198	0.675787
q076	243	3.781893	0.6786
q056	242	3.756198	0.816367
q057	242	3.739669	0.724781
q077	243	3.880658	0.608273
q078	243	3.687243	0.662547
q079	243	3.8107	0.672027
q080	243	3.839506	0.638764
q084	243	3.8107	0.696189
q085	243	3.855967	0.63623
q086	243	3.893004	0.63382
q087	243	3.90535	0.598977
q088	243	3.90535	0.585016
q089	243	3.880658	0.594531

## Annex 3: EFA results for Myanmar

**Table:**

Variable	Unrotated		Rotated	
	Factor1	Factor2	Factor1	Factor2
Because my reputation depends on it	0.1076	**0.4888**	− 0.128	**0.5362**
Because I receive appreciation for doing it	0.2837	**0.5858**	− 0.0024	**0.6519**
It brings pride to my family to know I’m contributing to malaria elimination	0.2599	**0.762**	− 0.1096	**0.8413**
It is a source of pride to participate in the franchise malaria programme	0.3648	**0.4771**	0.1284	**0.5389**
Because the program is interesting	**0.5618**	0.0549	**0.5217**	0.093
Because it is extremely important for my patients	**0.5246**	0.0044	**0.5095**	0.0361
I value the feedback about the effectiveness of my performance I receive from PSI about my contributions to PSI’s malaria programme	**0.6209**	0.0091	**0.6011**	0.047
My franchise malaria programme-related job duties, requirements, and goals are clear	**0.6617**	− 0.0624	**0.6751**	− 0.0281
Participating in the malaria programme gives me a feeling of accomplishment	**0.7231**	0.0403	**0.686**	0.0869
I feel I am contributing to malaria elimination in my community and country	**0.7509**	0.0236	**0.721**	0.0704
Participating in the programme makes me feel like I’m doing something worthwhile	**0.7645**	− 0.0151	**0.7527**	0.0293
There is satisfaction in a job well done	**0.8181**	− 0.101	**0.846**	− 0.0606
I am satisfied with the chances I have to learn new things	**0.8675**	− 0.1152	**0.9009**	− 0.073
I am satisfied with the chances I have to accomplish something worthwhile	**0.8553**	− 0.1501	**0.9056**	− 0.1116
I am satisfied with the chances I have to do something that makes me feel good about myself	**0.7218**	0.068	**0.6715**	0.1169
I am satisfied with the educational/training opportunities I get	**0.7462**	− 0.0868	**0.7691**	− 0.0495

## Annex 1: Description of Study Sites

PSI/Myanmar implements GEMS nationally in both high and low burden areas and through Sun Quality Health (SUN) network providers, Integrated Community Malaria Volunteers (ICMVs), and Non− Formal Private Outlet network members (POs). Between data collection and publication of results, the Artemisinin Monotherapy Replacement Network (AMTR) was renamed to Private Outlets, and the Community Health Services Provider (CHSP) was renamed Integrated Community Malaria Volunteers. In the text the networks are referred to as Pos and ICMVs. SUN providers are qualified physicians, typically General Practitioners (GPs) working in a clinic setting, whereas ICMVs, are similar to public sector community health volunteers and private outlet providers some of whom have medical training (e.g., auxiliary midwife). ICMVs are trained to provide community health interventions beyond malaria according to national policy. The PO network consists of mobile drug vendors, small drug shops, and general retail stores. ICMVs and POs are located in rural and peri-urban areas throughout the country and are responsible for the vast majority of testing and case detection. As part of participation in the program, SUN providers receive a maximum incentive of $16 per month, ICMVs receive a maximum of $10 per month, and POs receive a maximum of $5 per month. Through the engagement of these private sector providers, PSI/Myanmar tested 520,341 fevers in 2019, resulting in 4,388 confirmed cases detected. This accounted for approximately 14.2% of total fevers tested in country, with 8.3% of the national caseload detected through PSI’s networks [25]. The SUN providers detected 13.4% of all PSI networks’ positive cases, the ICMV channel detected 57.9%, and the PO channel detected 28.6%. Despite receiving fewer performance-based incentives (see Table

**Table 8:** Myanmar predicted external, internal, and financial motivation scores

		External motivation	Internal motivation	Financial motivation
*Provider characteristics*				
Overall	Overall mean score	0.000	0.001	− 0.002
	SD	0.710	0.262	0.798
	Min	− 1.971	− 1.207	− 0.579
	Max	**0.957**	0.183	3.094
Gender	Female	− **0.021**	− 0.004	0.037
	Male	0.040	0.009	− 0.077
Provider type	AMTR	**0.129**	− **0.028**	− 0.019
	CHSP	− **0.116**	**0.026**	0.014
	SQH	n/a	n/a	n/a
Region	Yangon	n/a	n/a	n/a
	Mandalay	**0.111**	− **0.066**	**0.402**
	Sagaing	− **0.607**	**0.072**	− **0.437**
	Taninthar	**0.410**	**0.064**	− **0.028**
	Shan	**0.353**	**0.109**	**0.400**
	Kachin	**0.112**	− **0.102**	− **0.116**
	Chin	**0.043**	− **0.050**	**0.494**
Education level	Monastery	**0.191**	**0.037**	0.040
	High school	− **0.139**	**0.006**	− 0.046
	Higher education	− **0.150**	− **0.103**	0.000
Provider age	Regression coefficient	0.008 **	0.001 ns	− 0.003 ns
Outcomes				
Attended PSI training	Yes	− 0.008	**0.013**	− 0.003
	Not yet	0.127	− **0.192**	0.017
How willing would you be to continue to keeping records and sharing them with the government?	Extremely unwilling	0.769	**0.333**	0.167
	Not willing	− 0.110	− **0.022**	− 0.042
	Neutral	− 0.142	− **0.296**	0.243
	Willing	− 0.028	− **0.041**	− 0.003
	Extremely willing	0.051	**0.078**	− 0.037
Imagine that all of PSI’s support for your practice ended tomorrow. How would you …	I wouldn't restock	**0.612**	**0.294**	**0.136**
	Receive from the government	**0.206**	− **0.080**	− **0.375**
	Buy from another source	− **0.205**	**0.078**	− **0.128**
	Other	**0.092**	**0.028**	**0.060**
Years in programme	Regression coefficient	0.027 ns	− 0.001 ns	− 0.015 ns

Bold scores indicate significant difference within group at the 5% level (ANOVA); significance of regression coefficients = * = 5%; ** = 1%; *** < 0.1%

8), the PO network tends to have the highest test positivity rate. SUN doctors have the second highest (despite lower testing rates than ICMVs and POs), likely due to their urban and peri-urban location and qualifications, which makes them more likely to test for confirmation of clinical diagnosis.

In Lao PDR, the PSI program operates in the five southern provinces and the low-burden, elimination-targeted north. In 2019 PSI supported 474 public–private mix (PPM) providers, consisting primarily of doctors and pharmacists, to test for, treat and report uncomplicated Pf and Pv cases (with ACT only). For their participation in the GEMS program, the PPM providers in Lao PDR receive a maximum incentive of $40 per month intended to cover things such as internet costs. In 2019, GEMS supported providers tested 73,754 fevers (13% of total fevers tested in the country) and detected 612 cases. A total of 9.2% of the national reported caseload in Lao PDR was detected through the PSI PPM network.

In Vietnam, the GEMS program operates in 4 provinces, primarily in the Central Highlands region. The GEMS network in Vietnam consists of private clinics (staffed by medical doctors), private pharmacies, community-based volunteers known as community malaria champions (CMCs), and fast-moving consumer goods shops (FMCGs). In agreement with some provincial authorities, PSI has trained pharmacies, CMCs, and consumer good shops to test and refer malaria cases. Providers in Vietnam do not receive financial compensation—only monthly/quarterly promotional material incentives valued at less than $43. In 2019, PSI’s 828 GEMS supported providers tested 28,421 fevers and detected 877 cases, accounting for 1.4% of the total fevers tested in country and 18.7% of the national reported caseload. The majority of cases in the PSI network are detected by clinics (74%), followed by pharmacies (13.6%). Incentive levels in each country are set by the program in the relevant PSI office based on program experience and where possible aligned with national implementation policy for example to align with NMCP supported community workers. Incentives are based on performance such as number of tests conducted each month and submission of surveillance reports.

## Abbreviations

AMTR: Artemisinin Monotherapy Replacement Program
CFA: Confirmatory Factor Analysis
CMC: Community Malaria Champion
CHSP: Community Health Service Provider
CHW: Community Health Worker
FMCG: Fast Moving Consumer Goods
GEMS: Greater Mekong Subregion Elimination of Malaria through Surveillance
GMS: Greater Mekong Subregion
ICMV: Integrated Community Malaria Volunteers
KMO: Kaiser–Meyer–Olkin
LMIC: Low Middle Income Countries
MMW: Mobile malaria workers
PO: Non-Formal Private Outlet
PPM: Public private mix
PSI: Population Services International
RDT: Rapid diagnostic test
SEM: Structural equation modelling
SQH: Sun Quality Health Network
SRMR: Standardized root mean squared
